# Robotic ureteral reconstruction for benign ureteral strictures: a systematic review of surgical techniques, complications and outcomes: Robotic Ureteral Reconstruction for Ureteral Strictures

**DOI:** 10.1186/s12894-023-01313-7

**Published:** 2023-10-12

**Authors:** Kunlin Yang, Karl H. Pang, Shubo Fan, Xinfei Li, Nadir I. Osman, Christopher R. Chapple, Liqun Zhou, Xuesong Li

**Affiliations:** 1 Department of Urology, Peking University First Hospital, Beijing, China; 2 Institute of Urology, Peking University, Beijing, China; 3 National Urological Cancer Center, Beijing, China; 4 Division of Urology, Department of Surgery, School of Clinical Medicine, The University of Hong Kong, Hong Kong, China; 5 Division of Urology, Queen Mary Hospital, Hong Kong, China; 6 Division of Surgery and Interventional Science, University College London, London, UK; 7 Section of Functional and Reconstructive Urology, Department of Urology, Royal Hallamshire Hospital, Sheffield Teaching Hospitals NHS Foundation Trust, Sheffield, UK

**Keywords:** Ureteral stricture, Robotic, Laparoscopic, Minimally invasive, Reconstruction

## Abstract

**Introduction:**

Robotic ureteral reconstruction (RUR) has been widely used to treat ureteral diseases. To summarize the surgical techniques, complications, and outcomes following RUR, as well as to compare data on RUR with open and laparoscopic ureteral reconstruction.

**Methods:**

Our systematic review was registered on the PROSPERO (CRD42022309364) database. The PubMed, Cochrane and Embase databases were searched for publications in English on 06-Feb-2022. Randomised-controlled trials (RCTs) or non-randomised cohort studies with sample size ≥ 10 cases were included.

**Results:**

A total of 23 studies were included involving 996 patients and 1004 ureters from 13 non-comparative, and 10 retrospective comparative studies. No RCT study of RUR was reported. The success rate was reported ≥ 90% in 15 studies. Four studies reported 85–90% success rate. Meta-analyses for comparative studies showed that RUR had significantly lower estimated blood loss (EBL) (P = 0.006) and shorter length of stay (LOS) (P < 0.001) than the open approach. RUR had shorter operative time than laparoscopic surgery (P < 0.001).

**Conclusions:**

RUR is associated with lower EBL and shorter LOS than the open approach, and shorter operative time than the laparoscopic approach for the treatment of benign ureteral strictures. However, further studies and more evidence are needed to determine whether RUR is more superior.

**Supplementary Information:**

The online version contains supplementary material available at 10.1186/s12894-023-01313-7.

## Introduction

Ureteral strictures can be malignant or benign in nature. Benign strictures are commonly caused by iatrogenic injury or trauma, urolithiasis, radiation and ischemia [1]. Treatment aims to relieve symptoms, prevent complications and renal failure. Management options include endoscopic treatments via dilatation or endoureterotomy. When ureteral strictures are refractory to endoscopic management, and patients do not wish to be nephrostomy or ureteral stent dependent, reconstructive surgery could be offered [1–3].

Ureteral reconstruction can be performed via an open, laparoscopic, or robotic approach [2, 3]. Principles include excision and anastomosis with or without a graft, or the use of flaps [3]. The technique used depends on the site and length of the stricture.

Open surgery is associated with large incisions, increasing blood loss, postoperative pain, and long length of stay (LOS) [4–6]. Although laparoscopic surgery is an alternative option, providing similar functional outcomes [7] with less peri-operative complications and shorter LOS [8], it is associated with a long learning curve [9].

Robotic-assisted (RA) laparoscopic surgery is associated with a shorter learning curve [9], and many studies reported that robotic ureteral reconstruction (RUR) is a safe and effective minimal-invasive approach for repairing the ureter, with high success rates and low complication rates [10–16]. However, RUR is performed in highly specialised centres, and outcome data are commonly from small cohort of patients with short follow-up.

In the past five years, there have been more studies published on RUR with larger cohort of patients and longer follow-up time [4, 10–24]. The aim of this systematic review is to summarize the surgical techniques, complications, and outcomes of RUR for benign strictures, and compare the available data on RUR versus open or laparoscopic ureteral reconstruction.

## Methods

### Search strategy

Our systematic review was registered on the PROSPERO database (CRD42022309364) and performed in accordance with the Preferred Reporting Items for Systematic Reviews and Meta-analyses (PRISMA) checklist. The PubMed, Cochrane and Embase databases were searched on 06-Feb-2022 (Supplementary 1-Search strategy). This was filtered for English articles and humans with no date restrictions.

### Study eligibility

A population (P), intervention (I), comparator (C), outcome (O), study design (S) (PICOS) framework defined the study eligibility. Studies were included if they fulfilled, (P): adult ≥ 18 years old patient with a benign ureteral stricture who underwent reconstructive surgery; (I): any reconstructive method, e.g., open, laparoscopic, or robotic surgery with or without the use of grafts or flaps; (C) any of the “intervention” methods listed above; (O) peri- and post-operative outcomes, including recurrence and reintervention rates. Complications, using the Clavien-Dindo (CD) classification; (S) randomised-controlled trials (RCTs) or non-randomised cohort studies.

Case reports, conference abstracts, reviews, letters, commentaries, and editorials were excluded. Non-English articles, studies with sample size less than ten cases, and studies including malignant cases were excluded. The studies of robotic pyeloplasty for treatment-naïve primary ureteropelvic junction obstruction (UPJO) and robotic repair for ureteroenteric strictures were excluded.

Articles were screened by two reviewers (KLY and KHP). Reference lists of included manuscripts were also screened for eligibility.

### Risk of bias assessment

The risk of bias (RoB) assessment of included studies was performed (KL and KHP) using the Newcastle-Ottawa scale RoB tool [25] for non-comparative cohort studies and non-randomised comparative studies.

### 2.4 Data extraction and analysis

The data extracted (KL, KHP, SBF, XFL) included, the number of patients, reconstructive technique, type of grafts and flaps used, baseline characteristics (age, stricture aetiology, stricture location, stricture length), operative time, blood loss, LOS, post-operative complications (e.g., fever, ileus, infection, anastomotic leak, fistula), CD grade, follow-up duration, recurrence rate, and reintervention rate.

As no RCT study was included in this review, we focused on a narrative synthesis. Comparative meta-analysis between robotic and open/laparoscopic approaches was summarized, where positive difference favours the open/laparoscopic approaches and negative difference favours the robotic approach. Statistical heterogeneity between studies was measured by the 95% confidence interval (CI) of mean differences (MD), P-value, and I^2^ (%) (a larger value for I2 represents a larger heterogeneity). When P-value > 0.1 and I^2^ < 50%, we used the fixed effect model. When P-value < 0.1 or I^2^ ≥ 50%, we used the random effect model. Meta-analyses were performed by using Review Manager 5.4.1 software (Cochrane Collaboration, Oxford, UK). A P-value < 0.05 was considered statistically significant (The P here for overall effect is different from the P in heterogeneity).

Continuous variables were described by the number of cases (n), mean, standard deviation (SD), median and interquartile range (IQR). In studies where mean value and SD were not reported, we used the formulas reported by Luo et al. [26] and Wan et al. [27] to calculate the estimated mean and the estimated SD.

## Results

### Quantity of evidence identified

**Fig. 1:**
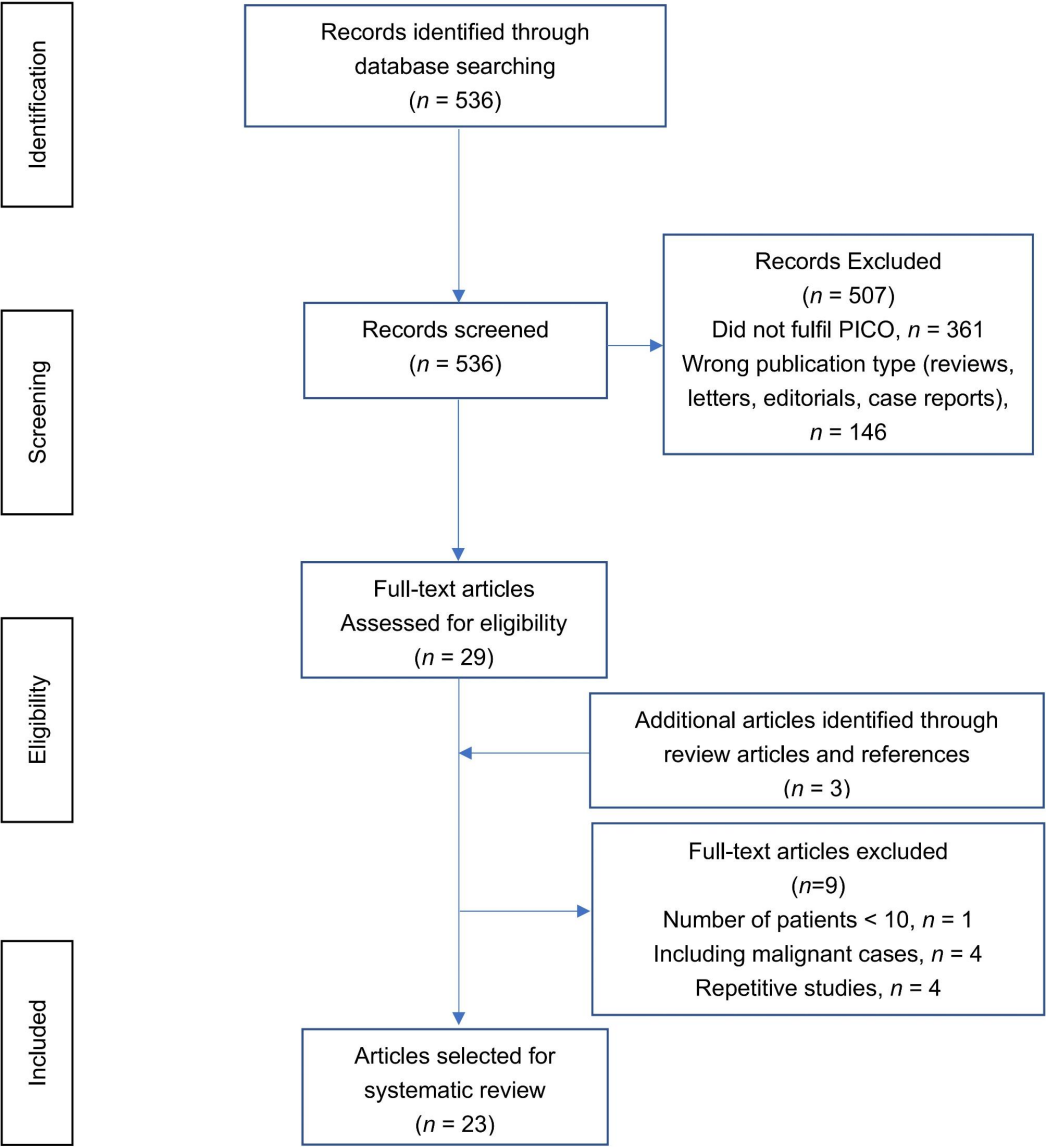
PRISMA flow diagram of the study selection process

A total of 536 articles were identified by our initial search, and 23 studies [4–6, 10, 12, 14, 15, 17, 20–24, 28–37] were included for analysis following abstract and full-text screening as shown in the PRISMA flow diagram (shown in Fig. 1). Overall, 996 patients (Ps) and 1004 ureters (Us) were analysed. Of these included studies, 13 were non-comparative studies, of which two were prospective [10, 28], and 11 were retrospective studies [14, 20, 21, 23, 29, 30, 32, 33, 35–37]. The remaining 10 were retrospective comparative studies [4–6, 12, 15, 17, 22, 24, 31, 34].

### 3.2 Characteristics of the studies included

Baseline characteristics of the patients including age, stricture aetiologies and locations, and type of surgery performed are presented in Table S1.

#### Aetiology associated with ureteral strictures

**Fig. 2:**
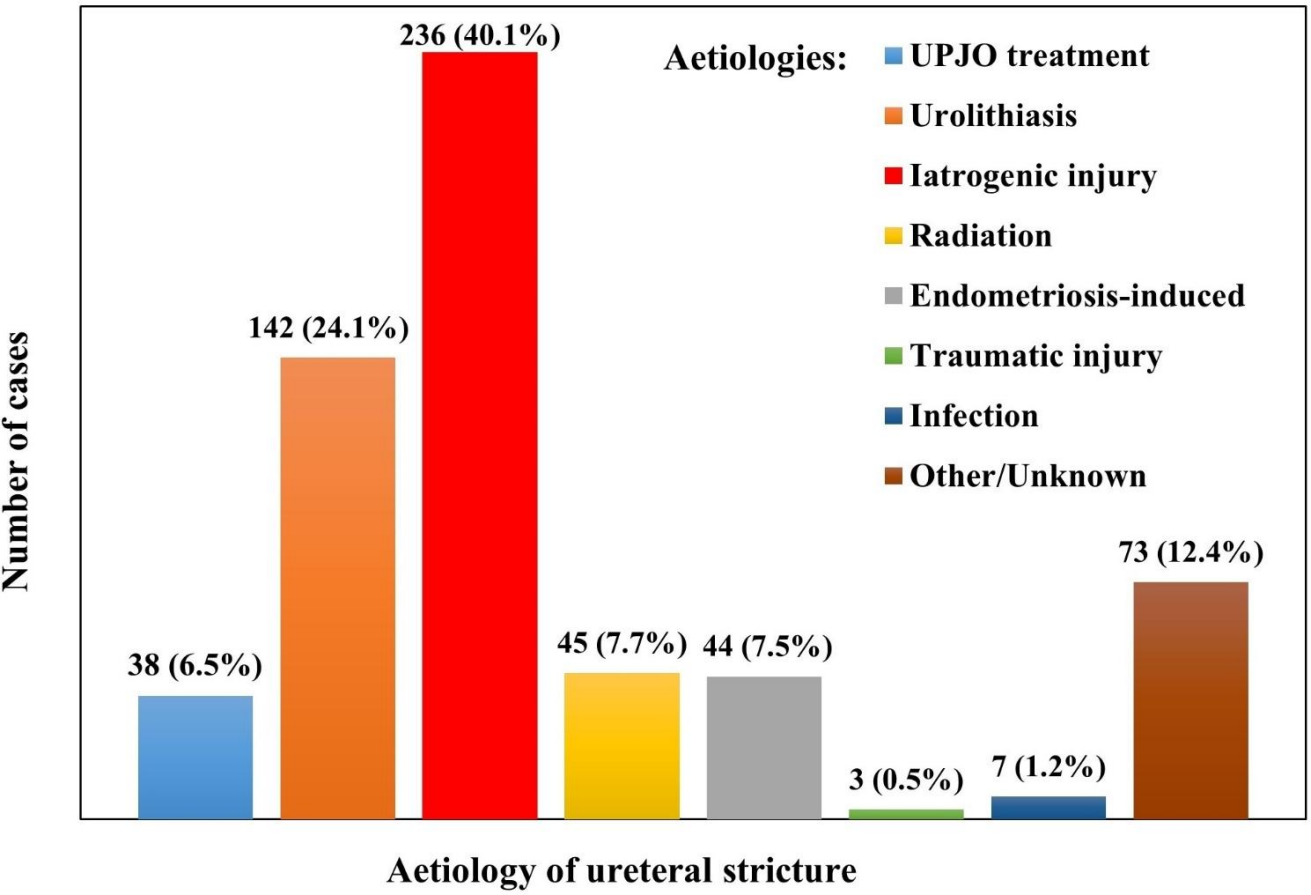
Aetiology associated with ureteral strictures

Aetiology data are available from 18 studies [5, 6, 10, 14, 20–22, 24, 28–37] (shown in Fig. 2). The aetiologies included: iatrogenic injury (n = 236, 40.1%); urolithiasis (n = 142, 24.1%); radiation (n = 45, 7.7%); endometriosis-induced (n = 44, 7.5%); UPJO treatment (n = 38, 6.5%); infection (n = 7, 1.2%); traumatic injury (n = 3, 0.5%); other/unknown (n = 73, 12.4%).

#### Location and length of the diseased ureters

In 699 ureters, 388 (55.5%) were proximal or UPJ, 214 (30.6%) were distal, 86 (12.3%) were middle, and 11 (1.6%) were both proximal and middle [4, 10, 12, 14, 17, 21, 22, 24, 28–30, 32–36]. The mean length of ureteral strictures ranged from 2.6 to 4.7 cm [5, 10, 17, 20, 28, 29].

#### 3.2.3 Types of surgery

There were 9 different types of surgery identified: lingual mucosa graft ureteroplasty (LMGU) [10], buccal mucosa graft ureteroplasty (BMGU) [14, 17, 28, 38], renal pelvic flap (RPF) [12], appendiceal flap ureteroplasty (AFU) [12], redo-pyeloplasty [17, 22, 23, 33], ureteroureterotomy [17, 22–24, 29, 31, 33–35, 37], appendix substitute (AS) [14], ureteral reimplantation (UR) [4–6, 14, 15, 20, 21, 23, 30–34, 36, 37], and ileal ureter replacement [14].

### Peri- and post-operative parameters

Data on blood loss, operative or console time, LOS, and follow-up time are detailed in Table S2.

### Complications

Complications following different forms of ureteral reconstruction are shown in Table S2. Thirteen studies reported complications following RUR [4, 6, 10, 14, 15, 22, 24, 31–34, 36, 37].

#### Clavien-Dindo grades

The incidence of CD I-II and CD IIIa/b were 0-30.8%, and 0-15.4% respectively [4, 6, 10, 14, 15, 22, 24, 31–34, 36, 37]. The most common complication type was fever. Other complications are listed in Table S2.

Only one study reported two cases of CD IV-V (n = 2/33, 6.1%) [14], while the others studies reported 0%. One patient who received ileal ureter replacement had an anastomotic bowel leak requiring a return to the operating room to repair the leak. The other patient had a myocardial infarction leading to death within 24 h of surgery [14].

### Efficacy of robotic ureteral reconstruction

#### Success rate

The success following RUR was mostly defined as no clinical symptoms and no radiological evidence of ureteral stricture. The success rate was ≥ 90% in 15 studies [4, 5, 10, 12, 17, 20, 28–30, 32–37], of which 9 studies reported a 100% success rate [4, 5, 12, 30, 32–36]. Four studies reported a success rate between 85.7% and 89.3% [14, 15, 22, 23]. The lowest success rate was 77.5% in a subgroup which had no preoperative ureteral rest [17].

#### Recurrence rate and reintervention rate

Three studies reported ureteral stricture recurrence rates, which ranged between 2.7% and 7.7% [21, 31, 37]. Reintervention rates of RUR were reported in three studies: 6.2-13.9% [21, 23, 29]. Details are demonstrated in Table S2.

### Comparison between interventions

#### Preoperative management: ureteral rest vs. no ureteral rest

Lee et al. [17] evaluated the effect of preoperative ureteral rest (absence of any kind of ureteral stent or tube across the ureteral stricture ≥ 4 weeks prior to RUR) on the final outcomes. The ureteral rest group had a median of 50mL EBL with 90.7% success rate compared to no ureteral rest group (75mL EBL, p < 0.001; 77.5% success rate, p = 0.03). The BMGU using rates were 20.1% for ureteral rest group and 37.5% for no ureteral rest group (p = 0.039).

#### Open vs. robotic

The comparative results between open and robotic approaches were reported in four studies including three studies on ureteral reimplantation [4–6] and one study on ureteroureterostomy and redo-pyeloplasty [22]. We performed meta-analyses for EBL, operative time, LOS, follow-up time and success rate.

##### Estimated blood loss

Four studies reported that the EBL with the robotic approach was reduced when compared to the open approach [4–6, 22]. Kozinn et al. reported that the mean EBL with RA-UR and Open-UR were 30.6mL and 327.5mL (P = 0.001) respectively [5].

**Fig. 3:**
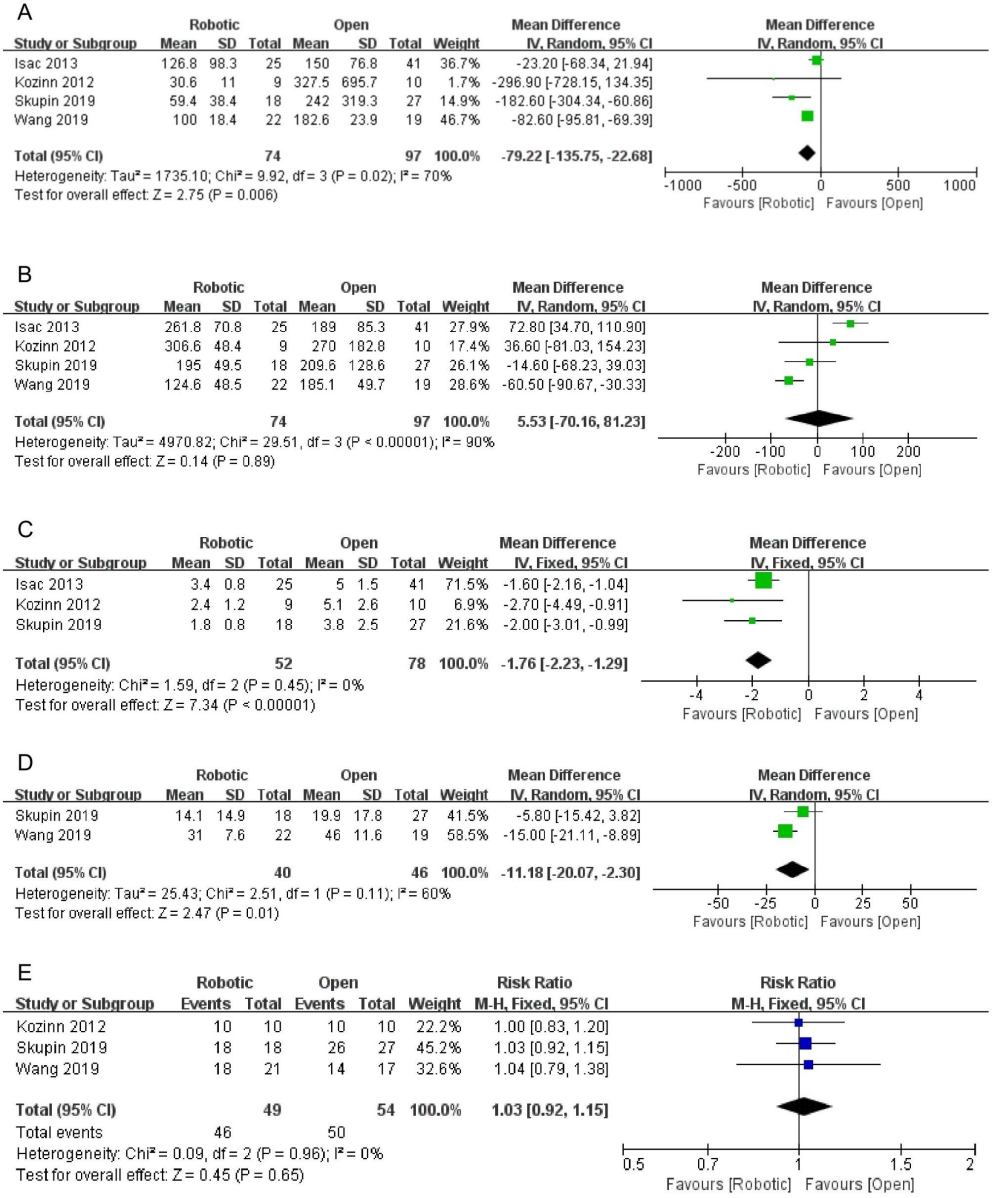
Forest plots of comparison between robotic and open ureteral reconstruction for estimated blood loss (**A**), operative time (**B**), length of stay (**C**), follow-up time (**D**) and success rate (**E**); SD, standard deviation; CI, confidence interval

Meta-analysis by a random effect model (Heterogeneity: P = 0.02, I^2^ = 70%) showed that EBL was significantly lower with the robotic approach than the open approach [4–6, 22]. Pooled mean difference (95% CI) was − 79.22mL [-135.75, -22.68] (P = 0.006, shown in Fig. 3. A).

##### Operative time

Two studies reported that robotic surgery was associated with a shorter operative time (mean, 124.6–195 min) than the open approach (185.1-209.6 min) [4, 22]. However, two studies reported the opposite results (robotic, 279-306.6 min vs. open, 200–270 min) [5, 6].

Meta-analysis by a random effect model (Heterogeneity: P < 0.001, I² = 90%) showed that operative time was not significantly longer with the robotic approach than the open approach [4–6, 22]. Pooled mean difference (95% CI) was 5.53 min [-70.16, 81.23] (P = 0.89, shown in Fig. 3. B).

##### Length of stay

Three studies reported that the median LOS after RA-UR (1.5-3 days) was shorter than Open-UR (3-5.1 days) [4–6].

Meta-analysis by a fixed effect model (Heterogeneity: P = 0.45, I² = 0%) showed that the LOS was significantly shorter with the robotic approach than the open approach [4–6]. Pooled mean difference (95% CI) was − 1.76 days [-2.23, -1.29] (P < 0.001, shown in Fig. 3. C).

##### Complications

Skupin et al. [4] reported that the complication (CD I-II) rate of RA-UR and Open-UR were 5.6% and 14.8% (P = 0.34), respectively. Wang et al. [22] reported that CD I-II complication occurred in 9.1% of RUR and, no CD III-V complication occurred. However, in the open group, CD I-II and CD IIIa/b complications accounted for 36.8% (P = 0.057) and 10.5% respectively. Isac et al. [6] showed that RA-UR group had an 8% complication rate and 9.7% in the Open-UR group (P = 0.81).

##### Follow-up time

Meta-analysis of two studies [4, 22] by a random effect model (Heterogeneity: P = 0.11, I² = 60%) showed that the follow-up time was significantly shorter for the robotic approach than for the open approach. Pooled mean difference (95% CI) was − 11.18 months [-20.07, -2.30] (P = 0.01, shown in Fig. 3. D).

##### Success rates

Skupin et al. [4] reported the success rates of RA-UR and Open-UR were 100% and 96.3% respectively. Kozinn et al. [5] reported that both approaches had a 100% success rate. In another study [22], robotic surgery had an 85.7% success rate compared with open surgery (82.4%).

Meta-analysis by a fixed effect model (Heterogeneity: P = 0.96, I² = 0%) showed that the robotic approach had a higher, but not a statistically significant success rate than the open approach (Risk ratio = 1.03, 95% CI: [0.92, 1.15], P = 0.65, shown in Fig. 3. E) [4, 5, 22].

#### Laparoscopic vs. robotic

Five studies reported the comparative results between laparoscopic and robotic approaches [12, 15, 24, 31, 34]. Since Cheng et al. [12] reported two groups (group 1: renal pelvic flap, group 2: appendiceal flap) between the robotic and laparoscopic approach, we independently included these two groups into the meta-analysis.

##### Estimated blood loss

Cheng et al. [12] reported a median EBL with RA-renal pelvic flap of 50mL (lap, 30mL), and RA-appendiceal flap of 75mL (lap, 50mL). Baldie et al. [34] reported the mean EBL with RA-ureteroureterostomy/UR was 171mL, while the EBL of laparoscopic UR was 150mL. However, Schiavina et al. [31] showed that robotic surgery had a less mean EBL (robotic, 47.2mL vs. lap, 91.2mL).

**Fig. 4:**
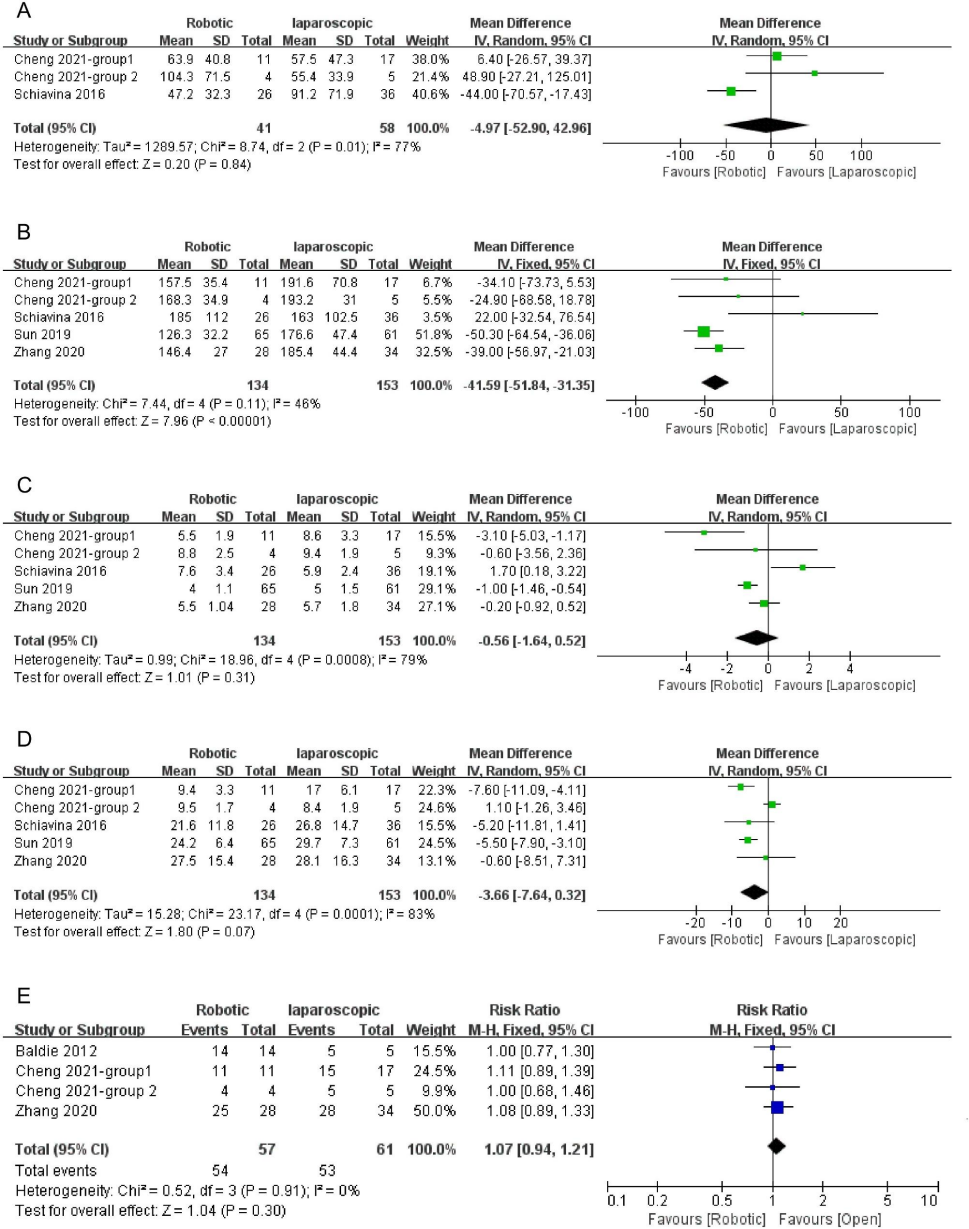
Forest plots of comparison between robotic and laparoscopic ureteral reconstruction for estimated blood loss (**A**), operative time (**B**), length of stay (**C**), follow-up time (**D**) and success rate (**E**); SD, standard deviation; CI, confidence interval

Meta-analysis by a random effect model (Heterogeneity: P = 0.01, I² = 77%) showed that EBL was insignificantly lower for the robotic approach than for the laparoscopic approach [12, 31]. Pooled mean difference (95% CI) was − 4.97mL [-52.90, 42.96] (P = 0.84, shown in Fig. 4. A).

##### Operative time

Except for one study that reported robotic surgery had a longer mean operative time (robotic, 185 min vs. lap, 163 min) [31], the other four studies reported that robotic surgery had a shorter mean operative time than laparoscopic surgery [12, 15, 24, 34].

Meta-analysis by a fixed effect model (Heterogeneity: P = 0.11, I² = 46%) showed that operative time was significantly shorter for robotic surgery than for laparoscopic surgery [12, 15, 24, 31]. Pooled mean difference (95% CI) was − 41.59 min [-51.84, -31.35] (P < 0.001, shown in Fig. 4. B).

##### Length of stay

In four studies, the mean LOS of robotic surgery (2.5–8.8 days) was shorter than laparoscopic surgery (2.7–9.4 days) [12, 15, 24, 34]. Only one study reported an opposite result (robotic, 7.6 days vs. lap, 5.9 days) [31].

Meta-analysis by a random effect model (Heterogeneity: P = 0.0008, I² = 79%) showed that LOS was shorter, but not statistically significant for the robotic approach than for the laparoscopic approach [12, 15, 24, 31]. Pooled mean difference (95% CI) was − 0.56 days [-1.64, 0.52] (P = 0.31, shown in Fig. 4. C).

##### Complications

The incidence of CD I-II complications was 4.6–7.7% following robotic surgery, and 5.6–16.7% following laparoscopic surgery [15, 24, 31, 34]. Two studies reported one (3.8% and 6.3%) case of CD IIIa/b complication in the robotic group [31, 34].

##### Follow-up time

Meta-analysis of four studies [12, 15, 24, 31] by a random effect model (Heterogeneity: P = 0.0001, I² = 83%) showed that the follow-up time was insignificantly shorter for robotic surgery than for open surgery [12, 15, 24, 31]. Pooled mean difference (95% CI) was − 3.66 months [-7.64, 0.32] (P = 0.07, shown in Fig. 4. D).

##### Success rate

Zhang et al. [15] reported the success rates after RA-UR and Laparoscopic-UR were 89.3% and 82.4% respectively. In another study [12], both robotic and laparoscopic surgery achieved 100% success rate when using the appendiceal flap technique. When using the renal pelvic flap technique, the success rate was 100% in the robotic group, and 88.2% in the laparoscopic group. Baldie et al. [34] showed that both techniques had a 100% success rate.

Meta-analysis by a fixed effect model (Heterogeneity: P = 0.91, I² = 0%) showed that the robotic approach had a higher, but not significant success rate than the open approach (Risk ratio = 1.07, 95% CI: [0.94, 1.21], P = 0.30, shown in Fig. 4. E) [12, 15, 34].

### Risk of bias assessment

Due to the lack of RCTs, the RoB assessments of the included studies were performed using the Newcastle-Ottawa scale RoB tool. Results are shown in Table S3. All comparative studies and non-comparative studies had high RoB due to the high selection bias.

## Discussion

### Principal findings

Here, we report the surgical approaches used in ureteral reconstruction for benign strictures. This systematic review extracted data from 23 studies (996 patients) on RUR using different surgical techniques. Due in part to the variety of surgical techniques and the lack of high-level RCT studies, it was difficult to draw on any firm conclusions.

In 18 studies reporting the aetiology of the ureteral strictures [5, 6, 10, 14, 20–22, 24, 28–37], the top two of the most common causes were iatrogenic injury (40.1%) and urolithiasis (24.1%). Therefore, it is important to take measures to reduce the risk of ureteral injury during ureteroscopic surgery, to avoid ureteral strictures. Meta-analyses showed that EBL and LOS were significantly decreased by the robotic approach compared to the open approach, and operative time was significantly shorter for the robotic approach than for the laparoscopic approach. Preoperative ureteral rest may improve the success rate of RUR and decrease the EBL and usage rate of BMGU [17].

The complication rates following RUR varied and was associated with the different type of surgical techniques performed. From our data, the robotic approach may have a lower complication rate compared with the laparoscopic or open approach [15, 24, 31, 34]. In addition, most studies reported a high success rate with RUR [4, 5, 10, 12, 17, 20, 28–30, 32–37].

**Fig. 5:**
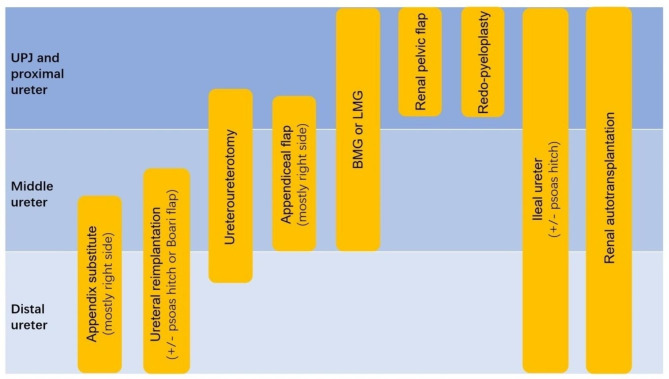
The surgical categories for ureteral reconstruction

All the main ureteral repair techniques can be performed robotically. A total of nine different surgical techniques were identified in this review. They can be divided into four major categories (shown in Fig. 5):

- *Excising the diseased tissue and shortening the distance to perform anastomosis directly*: Redo-pyeloplasty [17, 22, 23, 33], ureteroureterotomy [17, 22–24, 29, 31, 33–35, 37], UR [4–6, 14, 15, 20, 21, 23, 30–34, 36, 37].
- *Using a flap with blood supply or free graft to expand the ureteral lumen*: LMGU [10], BMGU [14, 17, 28], RPF [12], and AFU [12].
- *Complete replacement of the segmental or the whole ureter*: AS [14], ileal ureter [14].
- *Renal autotransplantation* [39, 40].

RUR has shown great benefits and has overcome the disadvantages of open and laparoscopic surgeries [33, 41, 42]. The robotic system provides the surgeon a magnified 3D vision and comfortable console platform to complete the intracorporeal operation safely and precisely [16]. More and more studies showed that RUR is a safe and effective minimal-invasive approach for ureteral repair with less blood loss, shorter hospital stay, higher success rates, and lower complication rates [10–16].

A 2018 systematic review by Kolontarev et al. [43] included 12 retrospective studies up to 2016 which reviewed the RUR literature and compared available data on robotic surgery versus open surgery. They reported that the EBL was significantly lower for RUR than for open surgery. Babbar et al. [44] reported a narrative review on different RUR techniques and concluded that ureteral reconstruction benefited from the robot with the fine tissue manipulation required, and the promise of improved cosmesis and minimal blood loss. In recent years, there have been more studies on RUR reported by different centres. Therfore, we summarized recent data in the literature focusing on the surgical outcomes of RUR for benign ureteral strictures, as well as comparative results on RUR versus open or laparoscopic ureteral reconstruction.

### Implication for clinical practice

The findings in this review would be helpful in offering treatment options for ureteral repair. Firstly, pre-operative ureteral rest may improve the success rate of RUR and decrease the EBL and usage rate of BMGU [17]. Removal of ureteral stent and placing nephrostomy have become a routine preparation for complex ureteral repair. However, further research is still needed to support this point.

We also highlighted that RURs using LMGU [10, 12], BMGU [13, 16, 17, 45], and AFU [12] are feasible and effective techniques for the upper and middle ureteral reconstruction.

As shown in Fig. 5, ileal ureter replacement [14] and renal autotransplantation [39, 40] may be the “last resort” options to salvage kidneys with complex ureteral strictures that are not amenable to in-situ reconstruction. Decaestecker et al. [39] reported that robotic renal autotransplantation (RRA) is a feasible and safe option for the selected patients with complex ureteral strictures. Compared with robotic ileal ureter replacement, RRA may require more skills including experience in robotic renal, vascular, and transplant surgery, which may limit its usage [40].

### Implication for future research

Most studies included in this review were retrospective or case series in nature, with relative low certainty of evidence. Up until now, no RCT on RUR has been reported. Although it can be difficult in surgical settings and patient selection, RCTs are needed to provide high-level of evidence.

In addition, there are no standardised success criteria for RUR. Some studies defined success as no clinical or radiologic evidence of recurrent stricture disease [5, 30]. Some studies defined success as no clinical symptoms and no radiographic obstruction [10, 13, 18, 46]. We must emphasize the importance of standardising the reporting of RUR outcomes.

In our opinion, wherever possible, the urinary tract should be reconstructed either using a renal pelvic flap, reimplantation or uretero-ureterostomy if feasible. In terms of substitution, where possible, it is best to avoid interposing bowel based on our past experience that this tends to lead to atonic segments which lead to dysfunction of the upper tracts. It is far better using onlay grafts of oral mucosa, either lingual or buccal, seems to be, in our view, the most appropriate option rather than interposition of bowel. Certainly, based on the evidence available, here it is not possible to make definitive comments.

### Strengths and limitations

This review has some strengths including the systematic approach, well-designed methodology, adherence to the PRISMA checklist and RoB assessment of individual studies which ensure that the included studies provided meaningful information. We performed a narrative synthesis to show the data of RUR and to reduce the risk of reporting inaccurate conclusions.

However, we must face some limitations due to the selection bias and large heterogeneity among studies. RUR is a large field including any robotic reconstructive surgery for any ureteral disease. Except for benign ureteral strictures, RUR has been wildly used for the treatment of congenital ureteral malformation, ureteral malignant diseases. Therefore, many studies on RUR were not included in this review according to our PICOS inclusion criteria.

## Conclusions

Most ureteral reconstructive techniques can be performed robotically, and RUR is becoming a useful approach and option for the treatment of benign ureteral strictures. RUR is associated with less EBL and shorter LOS than the open approach, and shorter operative time when compared to the laparoscopic approach. RUR has a higher success rate than open/laparoscopic surgeries.

### Electronic supplementary material

Below is the link to the electronic supplementary material.

Supplementary Material 1

Supplementary Material 2

Supplementary Material 3

Supplementary Material 4

## Data Availability

All data generated or analyzed during this study are included in this article and its online supplementary material. Further enquiries can be directed to the corresponding author.
