# Supplementary material for: Robotic ureteral reconstruction for benign ureteral strictures: a systematic review of surgical techniques, complications and outcomes: Robotic Ureteral Reconstruction for Ureteral Strictures
Source: BMC Urol. 2023 Oct 12;23:160. doi: 10.1186/s12894-023-01313-7 (PMC10571348; doi:10.1186/s12894-023-01313-7)
Supplement: Supplementary file 1 — Supplementary Material 1 [file 12894_2023_1313_MOESM1_ESM.docx]

**Search strategy**

Embase <1980 to 2022 Week 05>

Ovid MEDLINE(R) ALL <1946 to February 04, 2022>

1 (((Ureteric or ureteral or ureter) and (stricture or injury or obstruction or defect) and (ureteroureterostomy or pyeloplasty or ureteroplasty or reimplantation or vesicoureterostomy or psoas hitch or Boari flap or anastomosis or renal pelvic flap or ureterocalicostomy or buccal or lingual or graft or intestinal or ileal or appendix or interposition or tissue transfer or reconstruction) and (Robot or Robotic or Da Vinci)) not (children or paediatric or adolescent or infant)).af. not (case reports or editorials or letters or commentary or congress abstract or conference abstract or supplements or reviews or books or book chapters).pt. 810

2 limit 1 to english language 755

3 limit 2 to humans 689

4 limit 3 to yr="1990 -Current" 689

Embase <1980 to 2022 Week 05>

Ovid MEDLINE(R) ALL <1946 to February 04, 2022>

1 (((Ureteric or ureteral or ureter) and (stricture or injury or obstruction or defect) and (ureteroureterostomy or pyeloplasty or ureteroplasty or reimplantation or vesicoureterostomy or psoas hitch or Boari flap or anastomosis or renal pelvic flap or ureterocalicostomy or buccal or lingual or graft or intestinal or ileal or appendix or interposition or tissue transfer or reconstruction) and (Robot or Robotic or Da Vinci)) not (children or paediatric or adolescent or infant)).af. not (case reports or editorials or letters or commentary or congress abstract or conference abstract or supplements or reviews or books or book chapters).pt. 810

2 limit 1 to english language 755

3 limit 2 to humans 689

4 limit 3 to yr="1990 -Current" 689

5 remove duplicates from 4 536
